# ‘Quitlink’: Outcomes of a randomised controlled trial of peer researcher facilitated referral to a tailored quitline tobacco treatment for people receiving mental health services

**DOI:** 10.1177/00048674231181039

**Published:** 2023-06-23

**Authors:** Amanda L Baker, Kristen McCarter, Alyna Turner, Catherine Segan, David Castle, Lisa Brophy, Ron Borland, Peter J Kelly, Billie Bonevski, Donita Baird, Sacha Filia, John Attia, Stuart Szwec, Kerrin Palazzi, Sarah L White, Jill M Williams, Anna L Wrobel, Andrew Ireland, Karinna Saxby, Peter Ghijben, Dennis Petrie, Rohan Sweeney

**Affiliations:** 1 School of Medicine and Public Health, College of Health, Medicine and Wellbeing, University of Newcastle, NSW, Australia; 2 School of Psychological Sciences, College of Engineering, Science and Environment, University of Newcastle, Callaghan, NSW, Australia; 3 IMPACT Strategic Research Centre, School of Medicine, Barwon Health, Deakin University, Geelong, VIC, Australia; 4 Cancer Council Victoria, Melbourne, VIC, Australia; 5 Melbourne School of Population and Global Health, The University of Melbourne, Melbourne, VIC, Australia; 6 Centre for Complex Interventions, Centre for Addiction and Mental Health, Department of Psychiatry, University of Toronto, ON, Canada; 7 Social Work and Social Policy, School of Allied Health, Human Services and Sport, La Trobe University Melbourne, VIC, Australia; 8 Centre for Mental Health, Melbourne School of Population and Global Health, The University of Melbourne, Melbourne, VIC, Australia; 9 Melbourne School of Psychological Sciences, The University of Melbourne, Melbourne, VIC, Australia; 10 Illawarra Health and Medical Research Institute and the School of Psychology, University of Wollongong, Wollongong, NSW, Australia; 11 Flinders Health and Medical Research Institute (FHMRI), College of Medicine & Public Health, Flinders University, Bedford Park, SA, Australia; 12 Hunter Medical Research Institute, Newcastle, NSW, Australia; 13 Division of Addiction Psychiatry, Rutgers Robert Wood Johnson Medical School, New Brunswick, NJ, USA; 14 IMPACT – The Institute for Mental and Physical Health and Clinical Translation, School of Medicine, Deakin University, Geelong, VIC, Australia; 15 Orygen, Parkville, VIC, Australia; 16 Centre for Health Economics, Monash Business School, Monash University, Melbourne, VIC, Australia

**Keywords:** Tobacco treatment, smoking cessation, quitline, telephone counselling, peer worker, mental ill-health, severe mental ill-health, cost analysis

## Abstract

**Objective:**

The aim of this study was to test the effectiveness of a tailored quitline tobacco treatment (‘Quitlink’) among people receiving support for mental health conditions.

**Methods:**

We employed a prospective, cluster-randomised, open, blinded endpoint design to compare a control condition to our ‘Quitlink’ intervention. Both conditions received a brief intervention delivered by a peer researcher. Control participants received no further intervention. Quitlink participants were referred to a tailored 8-week quitline intervention delivered by dedicated Quitline counsellors plus combination nicotine replacement therapy. The primary outcome was self-reported 6 months continuous abstinence from end of treatment (8 months from baseline). Secondary outcomes included additional smoking outcomes, mental health symptoms, substance use and quality of life. A within-trial economic evaluation was conducted.

**Results:**

In total, 110 participants were recruited over 26 months and 91 had confirmed outcomes at 8 months post baseline. There was a difference in self-reported prolonged abstinence at 8-month follow-up between Quitlink (16%, *n* = 6) and control (2%, *n* = 1) conditions, which was not statistically significant (OR = 8.33 [0.52, 132.09] *p* = 0.131 available case). There was a significant difference in favour of the Quitlink condition on 7-day point prevalence at 2 months (OR = 8.06 [1.27, 51.00] *p* = 0.027 available case). Quitlink costs AU$9231 per additional quit achieved.

**Conclusion:**

The Quitlink intervention did not result in significantly higher rates of prolonged abstinence at 8 months post baseline. However, engagement rates and satisfaction with the ‘Quitlink’ intervention were high. While underpowered, the Quitlink intervention shows promise. A powered trial to determine its effectiveness for improving long-term cessation is warranted.

## Introduction

Although tobacco control measures, particularly when implemented comprehensively, have resulted in declines in smoking across many parts of the world, not all population groups have benefitted to the same degree (Dai et al., 2022). In Australia, tobacco smoking rates among people experiencing mental health conditions remain between two and five times higher than those without mental health conditions, with prevalence of smoking increasing with severity of mental health conditions (Australian Bureau of Statistics, 2018, 2021). Similar disparities in tobacco smoking rates have been reported in the United States (Dickerson et al., 2018; Szatkowski and McNeill, 2015). Although people experiencing mental health conditions are just as likely to make quit attempts and are even more likely to use cessation aids, they report less success in their quitting efforts (Twyman et al., 2014; Schwindt et al., 2017). Consequently, many people living with mental health conditions experience poorer quality of life (Dixon et al., 2007) and die prematurely from smoking-caused diseases (Bandiera et al., 2015).

There is accumulating evidence that people experiencing mental health conditions benefit from telephone-delivered interventions for health behaviour change, including tobacco smoking, though there is a paucity of evidence of cost-effectiveness (Baker et al., 2018). Proactive telephone counselling for tobacco dependence from quitlines has demonstrated effectiveness in the general population (Matkin et al., 2019). A recent review argued that quitlines could have high impact among people with mental health conditions who smoke, given their potential reach, utility and effectiveness when they are tailored to meet the special needs of this group (Berg, 2021). In existing randomised controlled trials (RCTs) in smokers with mental health conditions, tailoring of quitline interventions has included adding a mood management component among people with a past history of depression (Van der Meer et al., 2010); community-based group counselling focussing on smoking cessation among people attending community mental health centres (Morris et al., 2011) and more post-quit sessions for veterans attending mental health clinics (Rogers et al., 2016). Despite this accumulating evidence, few people who smoke, including those with mental health conditions, contact quitlines (Berg, 2021; Greenhalgh et al., 2022). To address this, we developed a tailored quitline intervention (known as ‘Quitlink’). Quitlink added dedicated counsellors plus 8 weeks combination nicotine replacement therapy (cNRT) to Quitline’s existing service in the state of Victoria, Australia, for people experiencing mental health conditions. Quitline’s existing service encourages use of mood management strategies that dually aid cessation and monitor nicotine withdrawal and medication side effects to help distinguish temporary withdrawal symptoms from psychiatric symptoms and to facilitate targeted treatment (Segan et al., 2017). We also engaged peer researchers, with their own experience of recovery from mental health conditions and tobacco smoking, and skills obtained from formal mental health peer support worker and research training (Byrne et al., 2021; Meagher and Stratford, 2018). Peer researchers facilitated recruitment, delivered the control intervention and proactively referred participants to quitline if randomly allocated to ‘Quitlink’ (Baker et al., 2019). A detailed description of the protocol that includes the rationale for a tailored approach has been previously reported (Baker et al., 2019).

This paper reports on the effectiveness and cost-effectiveness of Quitlink for cessation of tobacco smoking among people experiencing mental health conditions. It was hypothesised that Quitlink would be associated with higher rates of prolonged abstinence from tobacco smoking since the end of the treatment period (i.e. 6 months sustained abstinence) at 8-month post-baseline follow-up relative to the control condition. Secondary aims were to examine the effect of Quitlink on 7-day point prevalence abstinence and effects on cigarette consumption, quitting behaviours, other substance use, mental health and health-related quality of life (HRQL).

## Methods

### Design

We used a prospective, cluster-randomised, open, blinded endpoint (PROBE) design to compare a control condition with Quitlink.

### Inclusion/exclusion criteria

Participants were required to be smoking at least 10 cigarettes a day and accessing treatment or support from participating mental health agencies in Victoria (Mind Australia and St Vincent’s Mental Health). Exclusion criteria were current engagement in Quitline Victoria’s callback service; no ready access to a telephone; inability to complete informed consent and the screening survey; acute suicidality; contraindications to NRT and pregnancy. When online recruitment commenced (described below), inclusion criteria were expanded to include anyone in Victoria accessing treatment or support, including from their general practitioner, for a mental health, and alcohol or other drug use condition.

### Recruitment and consent

Study recruitment occurred between March 2019 and April 2021, with the 8-month post-baseline follow-up finalised in December 2021. As described in detail elsewhere (Baker et al., 2019; Sweeney et al., 2019), peer-facilitated recruitment strategies were adapted from face-to-face to direct mail (postcard) and online due to lower than expected recruitment, in part due to the impact of COVID-19 pandemic restrictions (Baker et al., 2022). Following provision of informed consent, baseline data were collected at enrolment within Research Electronic Data Capture (REDCap; Harris et al., 2009, 2019) via an iPad. Participants recruited online self-completed their baseline assessment at enrolment and follow-up assessments, with the exception of follow-up safety and diagnostic data which were collected via telephone. Participants received an AU$40 gift card on completion of baseline and each completed follow-up assessment.

### Randomisation

Procedures for blinding and randomisation were as described previously (Baker et al., 2019). Cluster randomisation was used in residential services where risk of contamination was higher, stratified by short- or long-term residence, with 1:1 allocation. Individual randomisation was used in community-based services where contamination risk was lower, via permutated block sizes of 4 and 6 to avoid incomplete blocks, stratified for site. Randomisation was managed by an independent statistician and the randomisation module was embedded in REDCap.

### Control condition

The control condition consisted of a peer researcher-delivered brief intervention (following baseline assessment and prior to randomisation) that included advice to quit, encouragement to use cNRT and to call Quitline, and provision of a Quit pack of written materials to motivate a quit attempt and support self-management. With consent, a letter was sent by the research team to health professionals and the participant nominated with information about the person’s trial participation, and a link to Australia’s smoking cessation guidelines for health professionals, including a list of medications affected by smoking.

### Intervention

The Quitlink intervention consisted all of the above and the following:

- A proactive referral to Quitline immediately following the brief intervention.
- Tailored and manual-guided Quitline counselling for people experiencing mental health conditions. A team of six Quitlink counsellors was trained to deliver the intervention. Specific to the Quitlink intervention, Quitlink offered continuity of care through having one counsellor allocated to each participant. Procedures for training and supervision of quitline counsellors and peer researchers have been reported previously (Baker et al., 2019). The counselling was based on cognitive behavioural principles and offered up to seven calls within an 8 week period (with additional calls allowed to deal with relapse crises and beyond if needed) and provision of written feedback to treatment providers at the end of the Quitline counselling programme.
- As in the control condition, with consent, a letter was sent to the person’s GP or psychiatrist. In addition, for the intervention condition, peer-reviewed articles that provide practical advice to assist doctors in helping people with mental illness to quit smoking were included.
- In addition, participants received a Quit Victoria brochure for carers and a Quitting Mood and Experiences Diary.
- Participants were offered up to 8 weeks of cNRT (patches plus an oral form of NRT [Baker et al., 2019]).

### Outcome measures

The complete list of outcome measures and assessment schedule can be found in Table 1. Key demographic, smoking (including 7-day point prevalence abstinence), alcohol and cannabis use, mental health and HRQL outcome measures are reported in this paper.

**Table 1.** Outcomes and assessment schedule.

	Baseline	2 months	5 months	8 months
Demographics	X			
Mental ill-health or AOD diagnosis				
Self-report – Have you ever received a diagnosis of a mental health or drug and alcohol problem?	X			
MINI (diagnostic interview) (Sheehan et al., 1998)		T	*	*
McLean Screening Instrument for Borderline Personality Disorder (MSI-BPD) (Zanarini et al., 2003)		T	*	*
Medications				
Current medications	X			
Smoking measures:				
Current smoking and quit attempts	X	X	X	X
7-day point prevalence abstinence		X	X	X
6-month prolonged abstinence (primary outcome)				X
Heaviness of smoking index (Heatherton et al., 1989; Kozlowski et al., 1994)	X	S	S	S
Tobacco types	X			
Expenditure on cigarettes	X	S	S	S
History (age first smoked)	X			
Social influences on smoking, e.g., lives with other smokers	X			
Cravings (Herd and Borland, 2009; Herd et al., 2009) – Currently, how often do you get strong cravings to smoke tobacco?	X	X	X	X
Situations not allowed to smoke		X	X	X
Goal	X			
Motivation to quit (Crittenden et al., 1994)	X	S	S	S
Confidence to quit – How confident are you that you can stop smoking for good in the next 2 months if you wanted to?	X			
Self-efficacy (Perkins et al., 2012) – How confident are you that you will not smoke at all tomorrow?		X	X	X
Products/services to help quit (including NRT, Quitline)	X	X	X	X
Nicotine replacement products (helpfulness, concern)	X			
Counselling preferences	X			
Mental health:				
Psychological distress (Kessler-10) (Kessler et al., 2003)	X	X	X	X
Substance use:				
Alcohol (AUDIT-C) (Bush et al., 1998)	X	X	X	X
Cannabis use with tobacco question	X	X	X	X
Cannabis (First question of CUDIT [Adamson et al., 2010])	X	X	X	X
HRQOL:				
EQ-5D (Herdman et al., 2011) + four AQol-8D (Richardson et al., 2014) psychosocial bolt-on questions^	X	X	X	X
Medications – NRT/cessation:				
Process measure (i.e. provided by intervention)		E		
Perceived support – GP, psychiatrist, other health professional		X		
Quitline use:				
Number, length, content and timing of and satisfaction with calls		E		
Service use				
Hospitalisations and other intensive health service use		X		X
Time off from work and usual duties		X	X	X
Financial stress questions adapted from Siahpush and Carlin (2006)	X	X	X	X
Therapeutic alliance:		X		
WAIT-3 (Warlick et al., 2018)		X#		
Peer worker brief intervention question		X		
PBS/MBS cost data				E

AUDIT-C: Alcohol Use Disorders Identification Test – Brief; AQoL-8D: Assessment of Quality of Life-8D; CUDIT-R: Cannabis Use Disorders Identification Test – Revised; CO: carbon monoxide; EQ-5D: EuroQoL 5-deimension; HRQL: Health-related quality of life; Kessler-10: Kessler Psychological Distress Scale; MINI: Mini International Neuropsychiatric Interview; NRT: nicotine replacement therapy; GP – general practitioner; WAIT-3: Working Alliance Inventory for Tobacco-3; MBS: Medicare Benefits Scheme; PBS: Pharmaceutical Benefits Scheme.

**Key**

* – If not captured at previous assessment.

E – Extracted data.

S – Current smokers.

# – For those who used Quitline.

T – Via telephone if a participant recruited online.

^ – To minimise participant survey burden as we moved away from face-to-face recruitment, we transitioned from capturing HRQL using the AQoL-8D instrument, which has strong psychosocial dimension properties, to the EQ-5D-5L plus four AQol-8D bolt-on questions (Baker et al., 2022). These can be used in combination to calculate HRQL utilities and has been shown to be comparable to the AQOL-8D (Chen and Olsen, 2020)

#### Primary outcome

Due to COVID-19 restrictions on in-person contact, the primary outcome was modified from biochemically (carbon monoxide, CO) verified to self-reported prolonged abstinence from tobacco smoking since the end of the treatment period (i.e. 6 months sustained abstinence, with no relapse, defined as seven or more days of continuous tobacco smoking and no reported tobacco smoking in the prior week) at 8-month post-baseline follow-up.

#### Secondary outcomes

Secondary outcomes were assessed by research assistants blinded to group allocation and intervention content via telephone. The assessment was abbreviated to reduce participant burden (removed assessment of smoking use motives [Cooper et al., 1992; Spencer et al., 2002], nicotine withdrawal symptoms [Toll et al., 2007] and changed HRQL instrument – see Table 1 note) as we transitioned from face-to-face to telephone then online recruitment due to COVID-19 restrictions.

Adverse events were coded according to the Medical Dictionary for Regulatory Activities codes (MedDRA^®^). MedDRA is the international medical terminology developed under the auspices of the International Council for Harmonisation of Technical Requirements for Pharmaceuticals for Human Use (ICH).

### Sample size and statistical analysis

Based on our previous study (Baker et al., 2006; Segan et al., 2011) and rates of cessation among those with more severe mental health conditions (Baker et al., 2006), we anticipated that prolonged abstinence would occur in 1% of the control arm vs 8% in Quitlink. To detect this effect with 80% power at *p* = 0.05, we required 134 participants per arm. We expected ~30% attrition and thus planned to recruit 382 smokers over 36 months.

All statistical analyses were completed using SAS v9.4 (SAS Institute Inc. Cary, NC, USA [SAS Institute, 2013]). Statistical significance was set a priori at *p* < 0.05.

Analysis of primary and secondary outcomes was carried out using a cluster randomised trial framework where the individuals recruited from community-based services were treated as clusters that contributed only one person, while individuals recruited from residential programmes were clustered together, i.e., called a split-plot design (Goulão et al., 2018). There was a total of 95 clusters (11 residential and 84 community-based participants of whom 14 were from St Vincent’s Hospital, 56 from Mind Centres for Mental Health and Wellbeing and 14 recruited online, equally distributed across both conditions). Of the 55 in the control condition, 42 participants were individual clusters and 13 were recruited across 6 other clusters. Of the 54 in the intervention condition, 42 were individual clusters and 12 were recruited across 5 other clusters.

Outcomes were modelled using generalised linear mixed models with linear regressions used for continuous outcomes, logistic regressions for dichotomous outcomes and ordinal logistic regression for ordinal outcomes. Mixed models handled the cluster and repeated measures at baseline, 2-, 5- and 8-month post-baseline follow-up periods. Within the models, individuals and clusters were modelled as random effects to account for the non-independence of measurements from the same individual and cluster, with group assignment and study time point – and their interaction – as fixed effects.

Mixed models allow for missing data under a missing at random assumption, but a sensitivity analysis using a worst-case scenario for smoking outcomes (intention to treat [ITT], missing imputed as smoking in the case of abstinence outcomes and not using NRT for the NRT use outcome) was also performed. In the case of continuous abstinence, participants who were smoking at 2 months post baseline were unable to meet the required criteria for abstinence at either the 5- or 8-month follow-up and as such were counted as not continuously abstinent even if not followed up.

When modelling continuous abstinence and 7-day point prevalence abstinence, study design effects (stratification and clustering) were not controlled for, due to lack of model convergence because of the high proportion of single-case clusters. As such, presented results for these outcomes should be interpreted cautiously.

Safety analysis used chi-square tests to compare percentage of participants in the Quitlink and control conditions experiencing adverse events by System Organ Class (SOC).

#### Cost-effectiveness analysis

As described previously (Sweeney et al., 2019), a within-trial cost-effectiveness analysis (CEA) was conducted, estimating the incremental cost-effectiveness ratios (ICERs) for (1) additional quit (primary outcome) and (2) quality-adjusted life years (QALYs) gained. Full details are presented in Supplementary Materials – CEA. QALYs were estimated by multiplying the HRQL utility scores by the number of months since last surveyed (2 or 3 months). The underpowered sample required some deviations from the planned protocol (discussed in Supplementary Materials) (Sweeney et al., 2019). Most notably, the within-trial analysis takes a more focused intervention implementation perspective, excluding indirect health resource utilisation costs incurred during the 8 months of follow-up. Costs associated with control and Quitlink were determined for each person using Quitline (staff time and call costs) and cNRT costs. We also included postcard mail outs as costs, as this is a likely strategy for reaching potential clients beyond the trial (see Baker et al., 2022). Costs were calculated in AUD 2021. Sensitivity analyses were conducted for both ICERs, and longer-term cost-effectiveness was also modelled (see Supplementary materials for further details and analyses).

## Results

**Figure 1.**
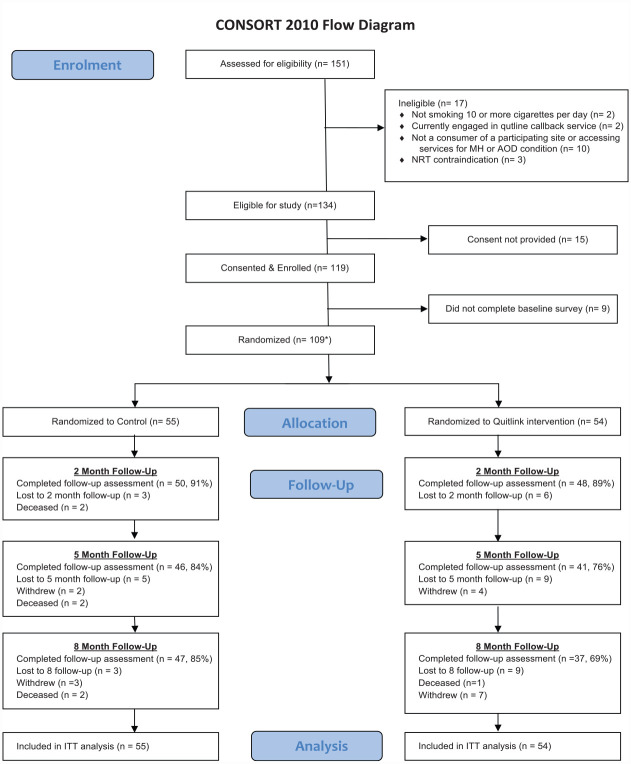
Consort diagram.

A total of 110 of our target sample of 382 people completed consent procedures and baseline assessments and were randomised (29 from face-to-face, 67 from direct mail postcard and 14 from online advertisement). One person subsequently withdrew from the study (including their baseline data), leaving a total sample of 109 people. Recruitment ended after 26 months in line with our grant timeline prior to achieving our target sample. As can be seen in Figure 1, significantly fewer participants assigned to the Quitlink condition completed the 8-month post-baseline follow-up compared to those in the control condition (*p* = 0.036). Participants who completed the 8-month follow-up were slightly older and somewhat more likely to have been recruited from a community-based service (Supplementary Table 1).

Demographic and baseline clinical variables of the two conditions are shown in Table 2. About half the sample was female, with an average age of approximately 45 years, and most were receiving disability support benefits. Most had a diagnosis of a psychotic disorder, about half consumed alcohol above national guideline recommendations, while a quarter had used cannabis in the previous month. Moderate-to-high tobacco dependence on the HSI (Heatherton et al., 1989; Kozlowski et al., 1994) was evidenced by mixed tobacco consumption with people smoking around 20 cigarettes per day. Although almost all had made a quit attempt at some time, with about half trying in the past year, confidence in quitting was low (see Table 2). As described earlier, when online recruitment commenced, inclusion criteria were expanded to include anyone in Victoria accessing treatment or support, including from their general practitioner, for a mental health, and alcohol or other drug use condition. However, 9 of the 14 participants recruited online reported an alcohol or other drug use condition (*n* = 7 had a co-occurring mental health condition).

**Table 2.** Baseline participant characteristics by intervention allocation.

Variable	Control	Quitlink	Total
	(*n* = 55)	(*n* = 54)	(*N* = 109)
Gender (female), *n* (%)	29 (52.7)	27 (50.0)	56 (51.4)
Age (years), M (SD)	43.56 (13.26)	46.26 (12.82)	44.89 (13.05)
Married/de facto, *n* (%)	9 (16.4)	7 (13.0)	16 (14.7)
Unemployed, *n* (%)	36 (65.5)	28 (51.9)	64 (58.7)
Receiving disability support benefit, *n* (%)	35 (63.6)	32 (59.3)	67 (61.5.0)
Left school at or before 16 years, *n* (%)	22 (40.0)	13 (24.1)	35 (32.1)
Supported residential accommodation, *n* (%)	13 (23.6)	12 (22.2)	25 (22.9)
Age started smoking (years), M (SD)	16.11 (4.34)	17.02 (7.03)	16.65 (5.83)
Types of tobacco used, *n* (%)			
Cigarettes	43 (78.2)	40 (74.1)	83 (76.1)
Pouch	24 (43.6)	30 (55.6)	54 (49.5)
Bulk	16 (29.1)	11 (20.4)	27 (24.8)
Butts left behind	10 (18.2)	15 (27.8)	25 (22.9)
Cigarettes per day, M (SD)	21.29 (9.99)	20.30 (9.56)	20.80 (9.75)
HSI addiction category, *n* (%)			
Low	6 (10.9)	7 (13.0)	13 (11.9)
Moderate	37 (67.3)	35 (64.8)	72 (65.1)
High	12 (21.8)	12 (22.2)	24 (22.0)
Quit attempt (ever), *n* (%)	45 (81.8)	51 (94.4)	96 (88.1)
Quit attempt in last year, *n* (%)	23 (41.8)	27 (50.0)	50 (45.9)
Currently trying to cut down, *n* (%)	46 (83.6)	41 (75.9)	87 (79.8)
Motivation to quit, *n* (%)			
Not at all	3 (5.5)	2 (3.7)	5 (4.6)
A little	2 (3.6)	4 (7.4)	6 (5.5)
Some	10 (18.2)	8 (14.8)	18 (16.5)
Very much	40 (72.7)	40 (74.1)	80 (73.4)
Main goal to quit smoking in next 2 months	36 (65.5)	34 (63.0)	70 (64.2)
Confidence in quitting in next 2 months, *n* (%)			
Not at all	19 (34.5)	14 (25.9)	33 (30.3)
Somewhat	13 (23.6)	13 (24.1)	26 (23.8)
Moderately	12 (21.8)	17 (31.5)	29 (26.6)
Very	3 (5.5)	7 (13.0)	10 (9.2)
Extremely	8 (14.5)	3 (5.5)	11 (10.1)
How helpful will NRT be in helping quit? *n* (%)			
Not at all	10 (18.2)	4 (7.3)	14 (12.8)
Somewhat helpful	21 (38.2)	23 (42.6)	44 (40.4)
Moderately helpful	11 (20.0)	16 (29.6)	27 (24.8)
Extremely helpful	13 (23.6)	11 (20.4)	24 (22.1)
Likelihood of using NRT products in the long term, *n* (%)			
Not at all likely	18 (32.7)	12 (22.2)	30 (27.5)
Somewhat likely	11 (20.0)	13 (24.1)	24 (22.1)
Moderately likely	14 (25.5)	12 (22.2)	26 (23.9)
Extremely likely	12 (21.8)	17 (31.5)	29 (26.6)
Preference for in-person or telephone counselling for smoking, *n* (%)			
Face-to-face	16 (29.1)	16 (29.6)	32 (29.4)
Telephone	20 (36.4)	21 (38.9)	41 (37.6)
No preference	19 (34.5)	17 (31.5)	36 (33.1)
K10, M (SD)	29.04 (8.33) (*n* = 54)	26.39 (8.05) (*n* = 49)	27.78 (8.27) (*N* = 103)
MINI diagnosis (psychotic disorder), *n* (%)	32 (65) (*n* = 49)	27 (62.8) (*n* = 43)	53 (64.13) (*N* = 92)
AUDIT C % excessive drinker, *n* (%) (available case)	22 (41.0) (*n* = 54)	28 (52.8) (*n* = 53)	50 (46.7) (*N* = 107)
Cannabis use, *n* (%)			
Never	39 (72.2) (*n* = 54)	41 (75.9) (*n* = 54)	80 (74.1) (*N* = 108)
Monthly or less	8 (14.8)	4 (7.4)	12 (11.1)
2–4 times a month	3 (5.6)	6 (11.1)	9 (8.3)
2–3 times a week	0 (0)	0 (0)	0 (0)
4 or more times a week	4 (7.4)	3 (5.6)	7 (6.5)
Always or nearly always mix tobacco with cannabis, *n* (%)	12 (80.0)	9 (69.2)	21 (75.0)
HRQOL (ITT), M (SD)	0.50 (0.20)	0.55 (0.20)	0.52 (0.20)

HSI: Heaviness of Smoking Index (Heatherton et al., 1989; Kozlowski et al., 1994); MINI: Mini International Neuropsychiatric Interview (Sheehan et al., 1998); K10: Kessler Psychological Distress Scale (Kessler et al., 2003); AUDIT C: Alcohol Use Disorders Identification Test-Brief (Bush et al., 1998); HRQL: Health-related quality of life

### Descriptive characteristics of interventions received

Of the 54 participants allocated to the Quitlink intervention condition, 48 agreed to proactive referral to quitline and 45 received Quitlink calls. However, 6 of the 55 participants allocated to the control condition (11%) contacted Victoria’s Quitline and received Quitline’s standard tailored counselling for people with mental health conditions (no cNRT or dedicated counsellor). Number and duration of and satisfaction with quitline calls and the peer researcher brief intervention are presented in Table 3. All participants in the Quitlink condition were interested in receiving free NRT.

**Table 3.** Number and duration of and satisfaction with quitline calls and the peer researcher brief intervention.

Characteristic	Condition	Variables		
Total number and duration of quitline calls and days from first to last call between baseline and 2-month follow-up assessment		Call number	Minutes	Days
		M (SD),	M (SD),	M (SD),
		Range	Range	Range
	Control	2.20 (1.30)	29.00 (15.75)	6.75 (4.11)
		1–4 (*n* = 5)	11–52 (*n* = 5)	3–12 (*n* = 4)
	Quitlink	5.20 (3.16)	173.27 (131.04)	40.05 (17.07)
		1–15 (*n* = 45)	13–673 (*n* = 44)	1–66 (*n* = 38)
Total number and duration of extra quitline calls received and days from the first to last extra call between the 2- and 8-month assessment dates		Call number	Minutes	Days
		M (SD),	M (SD),	M (SD),
		Range	Range	Range
	Control	2.67 (1.53)	64.33 (22.03)	60.00 (32.60)
		1–4 (*n* = 3)	39–79 (*n* = 3)	26–91 (*n* = 3)
	Quitlink	3.83 (3.26)	86.89 (123.05)	112.00 (56.40)
		1–12 (*n* = 18)	2–509 (*n* = 18)	56.00–245.00
Total number and duration of calls received from baseline to 8 months		Call number	Minutes	
		M (SD),	M (SD),	
		Range	Range	
	Control	3.33 (2.07)	56.50 (43.19)	
		1–7 (*n* = 6)	11–131 (*n* = 6)	
	Quitlink	6.73 (5.22)	208.86 (189.13)	
		1–22 (*n* = 45)	13–827 (*n* = 44)	
Total number of unsuccessful call attempts from baseline to 8 months		M (SD)		
		Range		
	Control	5.00 (5.10)		
		1–15 (*n* = 6)		
	Quitlink	10.85 (6.28)		
		2–27 (*n* = 48)		
Total number of calls and duration of calls to a health practitioner from baseline to 8 months		Call number	Call duration	
		M (SD),	M (SD),	
		Range	Range	
	Quitlink only	2.00 (0.82)	15.0 (8.03)	
		1–3 (*n* = 4)	4.39–23 (*n* = 4)	
	Response	Control*n* (%)	Quitlink*n* (%)	
Satisfaction with quitline (2 months)		(*n* = 4)	(*n* = 32)	
Counsellor provided quitting information and strategies that were relevant	Not at all	0 (0.0)	2 (6.3)	
	A little	1 (25.0)	3 (9.4)	
	Moderately	2 (50.0)	3 (9.4)	
	Mostly	0 (0.0)	4 (12.5)	
	Very much so	1 (25.0)	20 (62.5)	
Satisfaction with the service received from quitline	Not at all	0 (0.0)	3 (9.4)	
	A little	0 (0.0)	0 (0.0)	
	Moderately	2 (50.0)	2 (6.3)	
	Mostly	1 (25)	3 (9.4)	
	Very much so	1 (25)	24 (75.0)	
Would call the quitline in future if wanting help with quitting	No	1 (25.0)	6 (19.0)	
	Yes	3 (75.0)	25 (78.0)	
	Don’t know	0 (0.0)	1 (3.1)	
Would recommend quitline to others	Definitely not	0 (0.0)	2 (6.3)	
	Probably not	0 (0.0)	2 (6.3)	
	Maybe	0 (0.0)	1 (3.1)	
	Probably	2 (50.0)	2 (6.3)	
	Definitely	2 (50.0)	25 (78.1)	
Level of comfort discussing smoking over the phone	Not at all	0 (0.0)	1 (3.1)	
	Somewhat	1 (25.0)	1 (3.1)	
	Moderately	0 (0.0)	4 (12.5)	
	Mostly	0 (0.0)	6 (18.8)	
	Completely	3 (75.0)	20 (62.5)	
Helpfulness of conversation 2 months ago with peer worker in encouraging you to try to stop smoking or cut down	Not at all helpful	1 (2.5)	0 (0.0)	
	A little helpful	8 (20.0)	5 (13.2)	
	Moderately helpful	8 (20.0)	6 (15.8)	
	Very helpful	23 (57.5)	27 (71.1)	
		*n* = 40	*n* = 38	

As can be seen in Table 3, between baseline and 8 months, Quitlink callers who received proactive quitline calls received twice the number and duration of calls compared to the small number (*n* = 6) in the control condition who chose to contact quitline. Table 3 also shows that satisfaction with quitline counsellors was higher in the Quitlink condition. Of those who had a conversation with a peer researcher (*n* = 78), most participants (82%) also rated their conversation (brief intervention) with a peer researcher as moderately or very helpful in encouraging change. As can be seen in Table 4, 36% in the control condition and 48% in the intervention condition reported using NRT in the past week at 2-month post-baseline follow-up (*p* = 0.243).

**Table 4.** Smoking outcomes.

Measure	Assessment occasion	Control, *n* (%)	Quitlink, *n* (%)	OR (95% CI) between conditions	*p*
Continuous abstinence (*N* = 91) (available case^)	5 months (i.e. 3 months continuous)	1 (2)	6 (14)	8.33 [0.52, 132.09]	0.131
	8 months (i.e. 6 months continuous)	1 (2)	6 (16)	8.33 [0.52, 132.09]	0.131
7-day point prevalence abstinence (*N* = 102) (available case)	2 months	2 (4)	11 (22)	8.06 [1.27, 51.00]	0.027
	5 months	6 (13)	10 (24)	2.21 [0.52, 9.41]	0.284
	8 months	6 (13)	9 (24)	2.12 [0.48, 9.39]	0.32
Using NRT within the last week (ITT)	Baseline	11 (20)	10 (19)		
	2 months	18 (36) *n* = 50	23 (48) *n* = 48	1.80 [0.67, 4.81]	0.243
	5 months	16 (35) *n* = 46	14 (34) *n* = 41	0.96 [0.33, 2.82]	0.945
	8 months	11 (23) *n* = 47	8 (22) *n* = 37	0.96 [0.29, 3.23]	0.953
				Sensitivity analysis#	
				1.51 [0.58, 3.90]	0.395
				0.79 [0.28, 2.18]	0.643
				0.70 [0.22, 2.23]	0.54
Using NRT or pharmacotherapy (e-cigarettes or stop smoking medication) within the last week (ITT)	Baseline	14 (25)	14 (26)		
	2 months	23 (46) *n* = 50	24 (50) *n* = 48	1.17 [0.47, 2.89]	0.739
	5 months	19 (41) *n* = 46	17 (41) *n* = 41	0.92 [0.35, 2.45]	0.87
	8 months	15 (32) *n* = 47	8 (22) *n* = 37	0.57 [0.19, 1.73]	0.323
				Sensitivity analysis#	
				1.02 [0.43, 2.44]	0.965
				0.78 [0.31, 1.96]	0.589
				0.42 [0.15, 1.24]	0.116
HSI category (ITT)	Baseline	*n* = 55	*n* = 54	(*N* = 109)	
	Low	6 (10.9)	8 (14.8)		
	Moderate	37 (67.3)	34 (63.0)		
	High	12 (21.8)	12 (22.2)		
	Quit smoking	0	0		
	2 months	*n* = 46	*n* = 30	0.38 [0.11, 1.41]*	0.148
	Low	16 (34.8)	14 (46.7)		
	Moderate	21 (45.7)	13 (43.4)		
	High	9 (19.6)	3 (10.0)		
	Quit smoking	2 (4.0)	12 (25.0)		
	5 months	*n* = 36	*n* = 29	1.56 [0.38, 6.42]*	0.534
	Low	15 (41.7)	12 (41.4)		
	Moderate	15 (41.7)	14 (48.3)		
	High	6 (16.7)	3 (10.3)		
	Quit smoking	7 (15.0)	11 (27.0)		
	8 months	*n* = 37	*n* = 25	0.96 [0.23, 3.97]*	0.953
	Low	14 (37.8)	11 (44.0)		
	Moderate	14 (37.8)	12 (48.0)		
	High	9 (24.3)	2 (8.0)		
	Quit smoking	7 (15.0)	9 (25.0)		
Cigarettes per day (all participants) (ITT)	Baseline	21.31 (9.97)	20.48 (9.37)		
	2 months	15.96 (13.00) (*n* = 50)	10.33 (11.09) (*n* = 49)	−4.76 [−9.48, −0.04]	0.048
	5 months	15.05 (15.25) (*n* = 46)	10.98 (10.74) (*n* = 41)	−2.49 [−7.36, 2.38]	0.315
	8 months	13.53 (14.30) (*n* = 47)	11.24 (11.4) (*n* = 37)	−1.02 [−5.94, 3.90]	0.683
Cigarettes per day (Continuing smokers) (ITT)	Baseline	21.31 (9.97)	20.48 (9.37)		
	2 months	16.62 (12.84) (*n* = 48)	13.68 (10.81) (*n* = 37)	−3.12 [−8.02, 1.78]	0.211
	5 months	17.77 (15.04) (*n* = 39)	15.01 (9.83) (*n* = 30)	−0.19 [−5.43, 5.04]	0.942
	8 months	15.90 (14.23) (*n* = 40)	14.86 (10.90) (*n* = 28)	0.86 [−4.45, 6.17]	0.75

HSI: Heaviness of Smoking Index (Heatherton et al., 1989);

Odds ratios (ORs) and associated 95% confidence intervals (CIs) with missing data classified as non-abstinent.

# – Sensitivity analysis using the worst-case scenario (not using NRT) was performed in the case of missing follow-up.

* – Not including those who had quit.

^ – The available case analysis includes the *N* study participants whose smoking status (continuous abstinence) was measured at 8 months post-baseline follow-up, plus *N* participants lost to 8-month follow-up, but who had been assessed as a non-successful quit at 2 or 5 months post-baseline follow-up, confirming them as non-successful quits at 8 months under the study definition of a successful quit as 6 months continual abstinence (Quitlink = N and control = *N* = 91).

### Primary outcome

As seen in Table 4, in an available case analysis (see Table 4 footnote for definition), there was a large but not statistically significant difference in self-reported prolonged abstinence at 8-month post-baseline follow-up between the Quitlink (16%, *n* = 6) and control (2%, *n* = 1) conditions (OR = 8.33 [0.52, 132.09] *p* = 0.131, *n* = 91). After worst-case sensitivity analysis (ITT) with missing data treated as not continuously abstinent, the intervention effect at the 8-month timepoint remained large (OR = 6.72 [0.45, 100.27] *p* = 0.165, *N* = 109) and not statistically significant (Table 4). The number of people successfully quit was the same at 5-and 8-month follow-ups.

### Secondary outcomes

**Figure 2.**
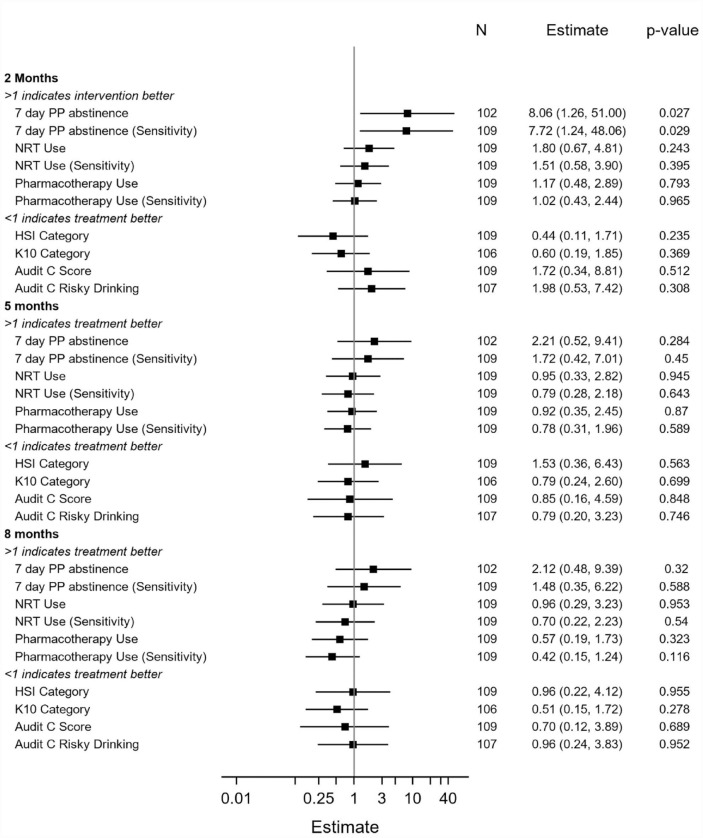
Secondary outcome modelling for categorical variables.

**Figure 3.**
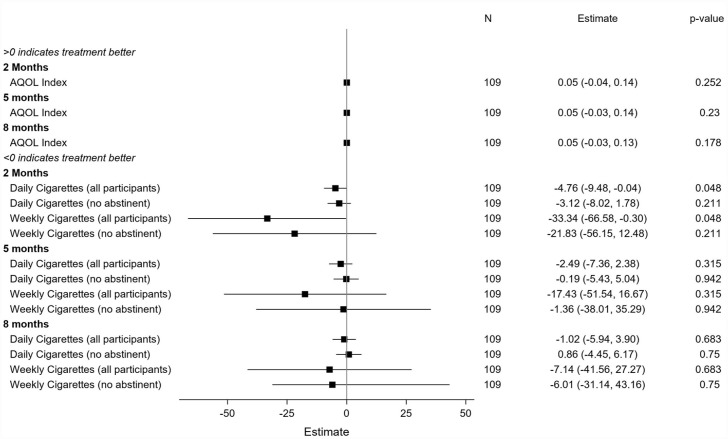
Secondary outcome modelling for continuous variables.

Data describing secondary outcomes are displayed in Table 4 (and Supplementary Table 2). Figures 2 and 3 present summaries of secondary outcome modelling. For categorical variables, ORs with 95% CIs, and p-values, are presented.

At the 2-month post-baseline follow-up (Table 4), there were significant differences in the odds of 7-day point prevalence abstinence between the Quitlink (11/49, 22.4%) and control (2/50, 4.0%) conditions (OR = 8.06 [1.27, 51.00] *p* = 0.027). There were no significant differences in this outcome at subsequent follow-ups. The overall interaction between intervention arm and study time period was not significant (*p* = 0.411), so this should be interpreted with caution. Table 4 shows that Quitlink participants achieved a statistically significant mean decrease in the number of cigarettes they smoked per day at the 2-month follow-up compared to control participants. There were no significant differences between conditions at subsequent follow-ups. There were no significant differences between intervention and control conditions in psychological distress (K10) or alcohol use (AUDIT) at any of the follow-up time points.

Over half of the participants (30 [56%] Quitlink and 31 [56%] control participants) reported experiencing at least one adverse event coded to SOC (Supplementary Table 3). During the trial, 86 serious adverse events (SAEs) were recorded (affecting 48 participants: 29 in the control group and 19 in the intervention group). Seven SAEs were rated by the study physician as possibly caused by the study and NRT, all psychiatric events (four events for three participants in the control arm; three events for three participants in the intervention arm). No differences were seen between the Quitlink and control conditions in the percentage of participants reporting adverse events or SAEs. ‘Psychiatric Disorders’ was the most frequently presenting SOC.

The estimated incremental cost per additional quit (6 months abstinence) at 8 months was between AU$9231 (*N* = 91, available case) and AU$11,333 (N = 109, worst case). The incremental cost per QALY over the 8-month period was estimated at AU$57,456 and has an approximately 48% chance of meeting a cost-effectiveness threshold of AU$50,000 AUD (Wang et al., 2018) in the short run (within-trial). When the available case sample was modelled over 50 years, the estimated cost per QALY gained was AU$54,361 (see Supplementary Materials for further details on assumptions, model parameters and analyses).

## Discussion

This trial did not find a significant difference on the primary outcome measure of self-reported prolonged abstinence at 8-month post-baseline follow-up between the Quitlink and control conditions. Point prevalence abstinence and cigarettes smoked per day favoured the Quitlink condition significantly at our first follow-up (2 months post baseline).

No secondary or sensitivity outcome showed any evidence of Quitlink being inferior. Further testing is required to determine if Quitlink could potentially be a relatively cost-effective way to help people experiencing mental health conditions quit smoking, at least in the short term (Barnett et al., 2008, 2015). Psychological distress scores did not worsen, and rates of psychiatric adverse events did not differ between conditions, supporting previous findings that addressing tobacco smoking does not worsen mental health (Anthenelli et al., 2016). Furthermore, any observed reduction in cigarette use may have important financial benefits for this generally economically disadvantaged population. Cigarettes in Australia are highly taxed (average pack is approximately AUD40), and 40% (42/109) of participants reported at baseline that their spending on cigarettes had resulted in having insufficient money for household essentials, such as food, in the previous month.

The small sample leaves significant uncertainty around estimates of intervention cost-effectiveness. Recruiting people with mental health conditions remains a real challenge, making conducting such trials expensive, probably requiring cooperation of a wider range of services. However, as they are a group that clearly need augmented assistance if they are to quit smoking, it is an investment that is likely worthwhile to determine what levels and nature of supports they need and to provide such services when there is sufficient evidence of their effectiveness. Supporting this investment, we present some evidence suggesting the Quitlink model may be cost-effective but more corroborating evidence is needed to be certain (Lightwood and Glantz, 1997; Rejas-Gutiérrez et al., 2017; Weber et al., 2021).

There are now two RCTs reporting sizeable prolonged self-reported abstinence rates following a quitline intervention among a sample with over half of participants experiencing psychotic disorders (Morris et al., 2011). However, sample size was limited in both studies. Future studies should investigate the effectiveness of quitline interventions among larger samples of people experiencing mental health conditions, including psychotic disorders.

Quitline counselling was acceptable in the present trial. Quitlink participants received more and longer calls than the few participants in the control condition who contacted quitline, and the Quitlink calls were rated more highly on satisfaction. It is likely that the additional training Quitlink counsellors received, the availability of a treatment manual and dedicated counsellors assigned to each participant all contributed to positive ratings. Both the Quitlink and control counsellors received regular supervision and feedback regarding their counselling, assuring quality across both conditions. This is consistent with our nested qualitative study that found compassionate support offered by the quitline counsellors was appreciated by participants and acknowledged how commonly this population experiences marginalisation and complex recovery trajectories (McCarter et al., 2022).

### Limitations

This study has several limitations. First and foremost, the study was underpowered due to recruitment challenges faced by peer researchers and exacerbated by COVID restrictions, as previously described (Baker et al., 2022). Due to this lack of power, we are unable to draw firm conclusions about the potential benefit of the Quitlink intervention.

There were significantly fewer people at the 8-month follow-up in the intervention group compared to the control group. It is possible that there was a difference in responsiveness to the intervention, with some being receptive to the more intensive intervention arm and others being actively put off, the latter being more likely to drop out. This would have inflated our effect size, except that the use of mixed models (which imputes missing data) and worst case and ITT sensitivity analyses mitigates such an effect. COVID restrictions also meant that CO verification of self-reported abstinence was not undertaken. Hence, our abstinence rates may be overestimated, potentially more so in the Quitlink condition as participants received more intervention and may therefore have been more inclined to feel social pressure to report quitting but as outcomes were assessed by people not engaged in clinical delivery, we think this unlikely. As we did not collect frequency of cNRT use, we were unable to relate this to effectiveness. A major strength of this study was its pragmatic design and minimal exclusion criteria, with findings likely representative of people receiving mental health services in the community.

## Conclusion

This study developed and tested peer researcher facilitated referral to a tailored quitline intervention (‘Quitlink’) for people receiving mental health services who smoke. The Quitlink intervention did not result in significantly higher rates of prolonged abstinence at 8 months post baseline. Participants had significantly higher 7-day point prevalence rates at 2 months and were more satisfied with the quitline service they received. Despite our lack of power, this provides important information about both effect size and acceptability for a subsequent trial. There is suggestive evidence that the intervention would be a relatively cost-effective way to help people experiencing mental health conditions to quit, at least in the short term.

## Supplemental Material

sj-docx-1-anp-10.1177_00048674231181039 – Supplemental material for ‘Quitlink’: Outcomes of a randomised controlled trial of peer researcher facilitated referral to a tailored quitline tobacco treatment for people receiving mental health services Supplemental material, sj-docx-1-anp-10.1177_00048674231181039 for ‘Quitlink’: Outcomes of a randomised controlled trial of peer researcher facilitated referral to a tailored quitline tobacco treatment for people receiving mental health services by Amanda L Baker, Kristen McCarter, Alyna Turner, Catherine Segan, David Castle, Lisa Brophy, Ron Borland, Peter J Kelly, Billie Bonevski, Donita Baird, Sacha Filia, John Attia, Stuart Szwec, Kerrin Palazzi, Sarah L White, Jill M Williams, Anna L Wrobel, Andrew Ireland, Karinna Saxby, Peter Ghijben, Dennis Petrie and Rohan Sweeney in Australian & New Zealand Journal of Psychiatry

sj-docx-2-anp-10.1177_00048674231181039 – Supplemental material for ‘Quitlink’: Outcomes of a randomised controlled trial of peer researcher facilitated referral to a tailored quitline tobacco treatment for people receiving mental health services Supplemental material, sj-docx-2-anp-10.1177_00048674231181039 for ‘Quitlink’: Outcomes of a randomised controlled trial of peer researcher facilitated referral to a tailored quitline tobacco treatment for people receiving mental health services by Amanda L Baker, Kristen McCarter, Alyna Turner, Catherine Segan, David Castle, Lisa Brophy, Ron Borland, Peter J Kelly, Billie Bonevski, Donita Baird, Sacha Filia, John Attia, Stuart Szwec, Kerrin Palazzi, Sarah L White, Jill M Williams, Anna L Wrobel, Andrew Ireland, Karinna Saxby, Peter Ghijben, Dennis Petrie and Rohan Sweeney in Australian & New Zealand Journal of Psychiatry

sj-docx-3-anp-10.1177_00048674231181039 – Supplemental material for ‘Quitlink’: Outcomes of a randomised controlled trial of peer researcher facilitated referral to a tailored quitline tobacco treatment for people receiving mental health services Supplemental material, sj-docx-3-anp-10.1177_00048674231181039 for ‘Quitlink’: Outcomes of a randomised controlled trial of peer researcher facilitated referral to a tailored quitline tobacco treatment for people receiving mental health services by Amanda L Baker, Kristen McCarter, Alyna Turner, Catherine Segan, David Castle, Lisa Brophy, Ron Borland, Peter J Kelly, Billie Bonevski, Donita Baird, Sacha Filia, John Attia, Stuart Szwec, Kerrin Palazzi, Sarah L White, Jill M Williams, Anna L Wrobel, Andrew Ireland, Karinna Saxby, Peter Ghijben, Dennis Petrie and Rohan Sweeney in Australian & New Zealand Journal of Psychiatry

sj-docx-4-anp-10.1177_00048674231181039 – Supplemental material for ‘Quitlink’: Outcomes of a randomised controlled trial of peer researcher facilitated referral to a tailored quitline tobacco treatment for people receiving mental health services Supplemental material, sj-docx-4-anp-10.1177_00048674231181039 for ‘Quitlink’: Outcomes of a randomised controlled trial of peer researcher facilitated referral to a tailored quitline tobacco treatment for people receiving mental health services by Amanda L Baker, Kristen McCarter, Alyna Turner, Catherine Segan, David Castle, Lisa Brophy, Ron Borland, Peter J Kelly, Billie Bonevski, Donita Baird, Sacha Filia, John Attia, Stuart Szwec, Kerrin Palazzi, Sarah L White, Jill M Williams, Anna L Wrobel, Andrew Ireland, Karinna Saxby, Peter Ghijben, Dennis Petrie and Rohan Sweeney in Australian & New Zealand Journal of Psychiatry
