# Supplementary material for: ‘Quitlink’: Outcomes of a randomised controlled trial of peer researcher facilitated referral to a tailored quitline tobacco treatment for people receiving mental health services
Source: Aust N Z J Psychiatry. 2023 Jun 23;58(3):260–76. doi: 10.1177/00048674231181039 (PMC10903138; doi:10.1177/00048674231181039)
Supplement: sj-docx-1-anp-10.1177_00048674231181039 – Supplemental material for ‘Quitlink’: Outcomes of a randomised controlled trial of peer researcher facilitated referral to a tailored quitline tobacco treatment for people receiving mental health services [file sj-docx-1-anp-10.1177_00048674231181039.docx]

**‘Quitlink’: Outcomes of a randomized controlled trial of peer researcher facilitated referral to a tailored quitline tobacco treatment for people receiving mental health**

**services**

**Cost-effectiveness analyses – Supplementary Materials**

#### Costs effectiveness analysis overview

A full cost-effectiveness analysis was planned^1^. However, the recruitment challenges and consequent underpowered study resulted in some deviations from the planned economic evaluation protocol. As planned, the within trial cost effectiveness analysis discussed in Section 2 below, estimates incremental cost effectiveness ratios (ICERs) for successful quits and quality adjusted life years (QALYs) gained at 8 months. However, a more focused intervention implementation perspective is taken, excluding within trial indirect health resource utilisation costs (discussed below). The longer term modelled analysis described in Section 3, estimates QALY ICERs and does incorporate longer term health resource utilisation (HRU) costs and cost savings associated with smoking cessation from the government perspective (including inpatient, outpatient and pharmaceutical resource use). Details of analysis methods, assumptions and deviations from protocol are discussed throughout below, including Section 4, which discusses within-trial included and excluded costs. Generally, there is significant uncertainty around cost effectiveness of the Quitlink intervention. However, there is sufficient evidence to suggest it could be a cost-effective smoking cessation intervention for people experiencing mental health conditions that should be investigated further through a larger well-powered trial.

1. ***Within trial cost effectiveness analysis***
   1. ***Description of methods***

The within trial cost-effectiveness analysis estimates the incremental cost per additional quit (self-reported 6 month prolonged abstinence) and quality adjusted life years (QALY) gained after 8 months, taking an intervention implementation perspective. Incremental quits are calculated using trial primary outcome data, first for the N=91participants who completed at 8 months follow-up or were observed “non-quitters” at 5 months and therefore by definition, non-quitters at 8 months (labelled as available case analysis), and then using the “worst case scenario” sample (N=109), where all losses to follow-up are assumed non-quitters.

Incremental QALY gains were estimated for participants who completed all health-related quality of life (HRQL) questions at baseline, 2, 5, and 8 months (N=72). HRQL was initially collected using the AQOL-8D instrument, which has strong psychosocial dimension properties (Richardson et al., 2014). To minimise participant survey burden as the study moved away from face to face recruitment, we transitioned to capture HRQL using the much briefer EQ-5D-5L plus 4 AQol-8D psychosocial bolt-on questions^2-4^. These can be used in combination to calculate HRQL utilities and has been shown to be comparable to the AQOL-8D ^4^. Total (QALYs) were estimated by multiplying the HRQL utility score by the number of months since last surveyed (2 or 3months). We applied the incremental per participant costs estimated foe the “worst case scenario” for this smaller sub-sample to estimate the within trial QALY ICER.

Given uncertainty of intervention arm costs and effects, we conducted bootstrapping analysis for each estimated mean ICER^5^. Taking a conservative approach, bootstrapping the Quit ICER uses the “worst case scenario” sample (N=109). The resulting ICER estimates were then plotted in cost-effectiveness planes (see Figures 1 and 2). Further, given some jurisdictions have implicit or explicit willingness to pay (WTP) thresholds for QALYs, we constructed a cost effectiveness acceptability curve, varying the WTP threshold for QALYs from $10,000 to $200,000 (see Figure 3).

Costs associated with the standard smoking care (Control) and Quitlink intervention group were determined for each individual. These included quitline call-related costs (staff time and phone company charges) and nicotine replacement therapy (NRT) costs. The quit support materials provided to the Control group were also provided to the Quitlink group, so associated printing costs are excluded. We assume beyond this trial setting that postcard mail-outs to clients of mental health service provider organisations will be the main recruitment strategy^2^. We calculate and include the estimated average mail-out cost per client recruited, using data from Quitline project records. We also include the cost of providing training to quitline counsellors, and average those costs over the anticipated number of clients recruited from scaling up to send postcards to the reported 61,101 adults who used mental health services in the state of Victoria^6^. We assume counsellors have some time-slack to absorb training time without affecting other tasks, and thus exclude their time cost to attend training. Note, the smoking status of people sent postcards in the trial was unknown and thus we assume the “postcard recruitment rate” observed in the trial applies to a scaled up Victoria-wide mail-out campaign (i.e. 66 recruited participants per 4210 unique addresses sent postcards = 1.6%). All costs are presented in 2021 Australian dollars (AU$). The detailed breakdown of included costs, costing assumptions and sources of evidence are presented in Section 4 after presentation of both the within trial and longer long term modelled ICERs.

Data on indirect health resource utilisation (HRU) were collected but excluded from the within trial analyses. The small study sample size, including only 48 participants who consented to release government linked administrative data on community-based health services (Medicare Benefits Scheme(MBS)) and pharmaceutical use (Pharmaceutical Benefits Scheme (PBS)), along with uneven loss to follow-up (more from the intervention group) created significant additional uncertainty. It was decided by the health economists involved that inclusion clouded the informative insights on short-term cost-effectiveness that could be made from an intervention implementation perspective. Generally speaking, there were no statistically significant differences in health resource utilisation between groups at 8 months. Section 4 below discusses this further and presents descriptive analyses of collected within trial data.

- 1. ***Results of within trial cost-effectiveness analysis***

Table 1 presents the total average costs, incremental treatment effects and estimated ICERs. As expected, Quitlink was associated with significantly larger intervention implementation costs of just over $1,000 (P<0.001) for both the available (N=91) and worst case scenario (N=109) samples. As discussed in the main paper, there was a large but non-significant difference in successful quits rates (0.141) favouring the Quitlink intervention. The estimated incremental cost per additional quit at 8 months was $9,231 (available sample) and up to $11,333 (worst case scenario sample). This is comparable to the short-term cost effectiveness in Barnett et al^7^ of US$11,496 per successful quit at 18 months, who compared a smoking cessation program given in an outpatient care setting in the USA for smokers with depression. When compared to a brief care comparator, their active intervention (including psychological counselling, NRT, and bupropion) increased successful quits after 18 months by 5.5% (p < 0.05) at a cost of USD11,496 per successful quit. Figure 1 suggests there is reasonable stability around incremental cost and incremental quit estimates from bootstrapping. With regards to QALYs, there was a small and non-significant difference between groups after 8 months (p=0.51), producing an estimated QALY ICER of $57,456 per QALY gained from Quitlink. Figure 2 presents the bootstrapping results. Assuming a WTP threshold of AU$50,000 ^8^, the distribution of the estimated change in costs and QALYs (Figure 3) indicates that slightly less than half (approx. 48%) of the point estimates are below the WTP threshold.

#### Table 1 – Total average costs, quits & QALY ICERs after 8-months.

| **Panel A – incremental cost per quit at 8 months** | | | | | | |
| --- | --- | --- | --- | --- | --- | --- |
|  | **Group** | **Cost (AU$)** | **Difference in costs** | **Quits** | **Difference in quits** | **ICER** |
| Available Case  (N=91) | Control (N=49) | $32.67 | $1,130.33 | 0.02  (1/49) | 0.122 | $9,231.03 |
|  | Quitlink  (N=42) | $1,163.00 |  | 0.14  (6/42) |  |  |
| Worst case scenario (N=109) | Control (N=55) | $29.11 | $1,054.00 | 0.018  (1/55) | 0.093 | $11,333.33 |
|  | Quitlink  (N=54) | $1083.11 |  | 0.111  (6/54) |  |  |
| **Panel B – incremental cost per QALY over 8 months** | | | | | | |
|  | **Group** | **Cost (AU$)** | **Difference in costs** | **QALYs** | **Difference in QALYs** | **ICER** |
|  | Control  (N=40) | $29.11 | $1,054.00 | 0.379 | 0.018 | $57,456 |
|  | Quitlink  (N=32) | $1083.11 |  | 0.397 |  |  |

**Notes:** QALY=quality-adjusted life year. ICER=Incremental Cost-effectiveness Ratio. QALY results show average values for sample with responses at each time point (Control=40 complete cases, Quitlink=32 complete cases). The worst-case scenario average costs are applied to the smaller sample (N=72) included in the QALY ICER analysis.

***Figure 1 – Distribution of incremental costs and incremental quits for Quitlink group relative to Control group – bootstrapping results.***
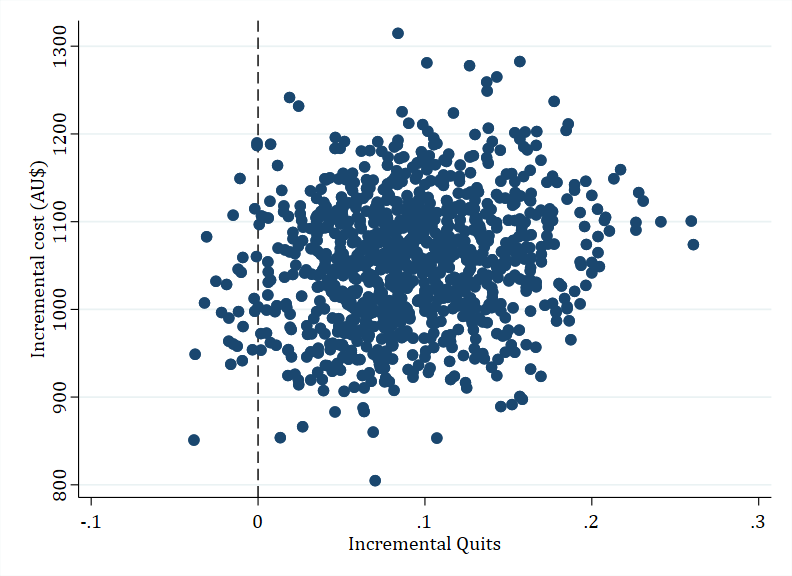


**Notes: Results show average incremental costs and quits for Quitlink vs Control group for 1,000 bootstrapped iterations for the worst case scenario sample (N=109).**

#### Figure 2 – Distribution of incremental costs and incremental QALYs for Quitlink group relative to Control group – bootstrapping results.

**Notes: QALY=quality-adjusted life year. WTP=willingness-to-pay threshold. Results show average incremental costs and QALYs for Quitlink vs Control group for 1,000 bootstrapped iterations.**

#### Figure 3 – Within-trial cost-effectiveness acceptability curve

**Notes: Figure shows probability of being cost effectiveness given different WTP thresholds for the estimated intervention costs.**

1. ***Modelling longer term costs and benefits***
   1. ***Description of methods***

The Smoking Duration and Intensity model was built to simulate the likely health outcomes and healthcare costs for an Australian cohort of smokers who are currently undertaking a quit attempt. It is a state-transition patient simulation model, consisting of health states related to smoking-status (smoker and ex-smoker) and smoking-related diseases (Chronic Obstructive Pulmonary Disease, lung cancer, acute myocardial infarction, and stroke). The four smoking-related diseases in the model capture the majority (over 60%) of smoking related mortality.

The model simulates lifetime outcomes for a hypothetical cohort of smokers who make a quit attempt, where baseline characteristics (age, gender, pack-years, smoking intensity, education, income and current/prior smoking related disease) influence the success of treatment, rates of relapse and future quit attempts, and the risk of future health events. Figure 4 presents the model structure.

Figure 4: Version 1.0 Smoking Duration and Intensity model structure (simplified)


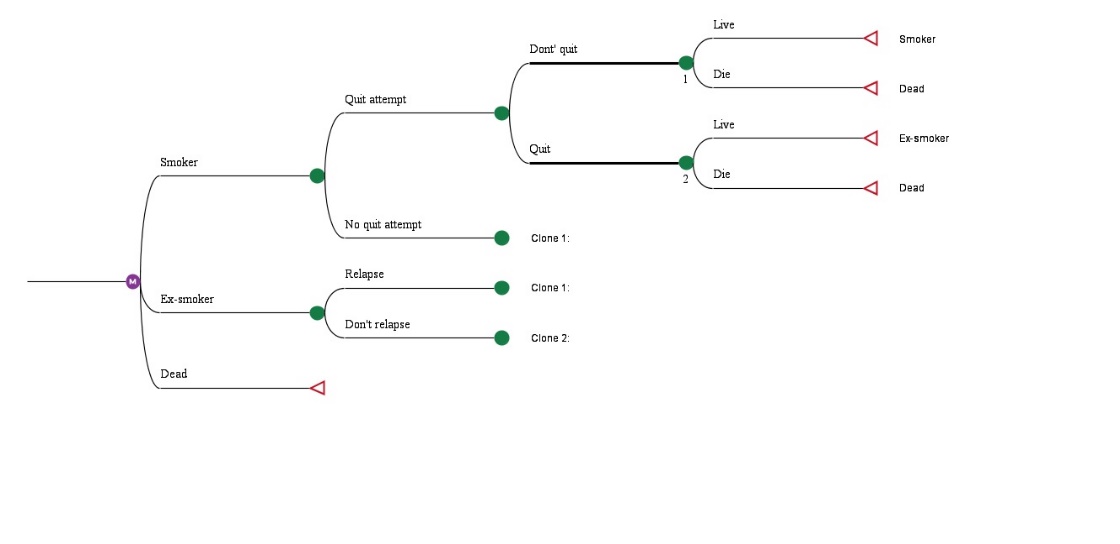

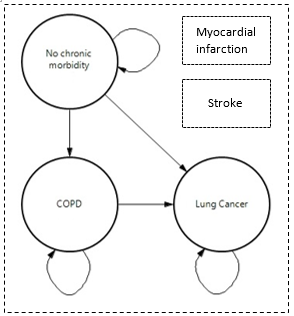


All people enter the model as a smoker and make a quit attempt in their first cycle. Conditional on survival, people who successfully ‘quit’ smoking (defined as 6-months continuous abstinence) transition to the ex-smoker health state in the subsequent cycle whereas people who do not quit remain as a ‘smoker’. In subsequent cycles, smokers have the chance to make additional quit attempts every 6 months and ‘ex-smokers’ have the chance to relapse.

To model smoking related disease, everyone is also assumed to enter the model in one of three chronic disease states (‘no chronic morbidity’, ‘COPD’ or ‘lung cancer’). Patients without a chronic illness are at risk of developing either COPD or lung cancer. The COPD health state is further divided by severity and patients have the chance to experience worsening COPD systems each cycle. To simplify the model structure and parameters, the model does not allow COPD diagnosis or progression after a lung cancer diagnosis. In addition, all patients remain at risk of having an AMI or stroke each cycle, which are modelled as transient events.

Version 1.0 of the model is setup to estimate the cost-effectiveness of smoking cessation treatments in the Australian context, with parameter estimates based on Australian data where available. The model population was drawn to reflect the baseline characteristics of patients enrolled in the Quitlink trial, with costs and quit rates for the index quit attempt informed by the within trial cost-effectiveness analysis.

To extrapolate costs and outcomes beyond the trial period, the model uses parameters from the literature. Rates for relapse and subsequent quit attempts by participant characteristics were estimated from the Household, Income and Labour Dynamics in Australia (HILDA) dataset and the Australian National Drug Strategy Household Survey^9^. These rates of relapse and subsequent quit attempts reflect the general smoking population. Examination revealed the HILDA panel includes only a small subsample of people with indicators of worse mental health (e.g. poor mental health domain scores on the SF-36 HRQL instrument) who were also smokers at some point and there were no identified differences in smoking relapse rates nor subsequent quit attempts compared to the general smoking sample. Given the lack of model parameter datapoints for Australians with severe mental illnesses (further research needed), model estimates should be treated with some caution and viewed as indicative only.

Given Quitlink treatment effect uncertainty, the model takes a simple approach of assuming one-off Quitlink intervention use. Quit rates and costs for subsequent quit attempts reflect the average for smoking cessation treatments used (nicotine replacement therapy, varenicline and unassisted) in Australia based on the National Drug Strategy Household Survey. Absolute quit rates associated with each treatment choice by smoking intensity were estimated based on results of a network meta-analysis and underlying quit rates observed in a recent Australian clinical trial^10^. To model the incidence of smoking-related disease, rates by patient characteristics (including pack-years and years smoke-free) were estimated from the literature^11-24^. Excess mortality rates associated with smoking-related diseases and caused-eliminated mortality rates (i.e. other causes) by patient characteristics were estimated using Australian life tables and Australia registry data^25, 26^. Further details of the model are forthcoming (Pharmaceutical Benefits Scheme, Report forthcoming – details in References below)^27^.

- 1. ***Results of modelled longer term costs-effectiveness analysis***

When the Available Case sample (N=91) are modelled over 50 years and applying 3% discounting to future costs and benefits, one run of 600,000 potential Quitlink smokers estimated Quitlink to be AU$913 dollars more expensive than Control and improve QALYs by 0.0168, producing an ICER of AU$54,361 per QALY gained. The estimated ICERS for 0% and 5% discounting of costs is $16,339 and $102,603 per QALY gained. Comparing findings across studies that model longer term cost effectiveness is difficult. Model parameters and assumptions vary greatly, including some do not account appropriately for relapse rates and costs associated with subsequent quit attempts. There is sufficient evidence to suggest that Quitlink may be cost effective in the longer run, warranting further trial-based and model-based investigations.

1. ***Details of included and excluded costs and assumptions***

Table 2 details the unit costs and assumptions of costs included in the within-trial CEA described in Section 2. Table 3 details intervention-based NRT expenditure by NRT type.

Tables 4 & 5, provides descriptive analyses of excluded within-trial HRU costs. Specifically, self-report survey questions for inpatient hospital and stays in community-care mental health facilities (Table 4), and linkage data to the Services Australia data on Medicare (Medicare Benefits Scheme (MBS)) and Pharmaceutical Benefits Scheme (PBS) for community-based non-inpatient health service and pharmaceutical costs (Table 5). Only 48 participants consented to release either their Medicare and/or PBS data, many more of whom in the control group were Medicare concessional card holders (p=0.05), eligible for larger government subsidies. Both sources of HRU data were analysed and generally speaking there was no significant differences across categories after 8 months follow-up. The intervention group reported significantly fewer mental health related emergency department visits over 8 months of follow-up (Table 4). However, the small sample, higher drop-out rate in the intervention group plus no observed difference in K10 scores raises concern about including this high-expense cost-offset. Mean monthly Government PBS and out-of-pocket Medicare expenditures was much larger (though non-significant) in the Quitlink group after 8 months (Table 5). However, these expenditure differences were present during the (on average) 21 months of health resource use data prior to randomisation in the study. These data uncertainties informed the decision to take a more focused intervention implementation perspective. Excluding within trial indirect health resource utilisation costs gives more useful insights for researchers/funders considering implementing a larger RCT or real-world trial.

**Table 2. Included costs, assumptions and sources of evidence**

| **Cost types** | **Unit costs & assumptions** | **Sources of evidence** |
| --- | --- | --- |
| Costs of training Quitline staff | A one-day, 8-hour training would be delivered (each year) by a clinical psychologist costed at the Australian Psychological Society recommended rate $240/hour including on-costs for a total cost of $1,920. A 40-page manual would be provided at a total printing cost of $12 ($0.10/page printed). To estimate the average cost per client recruited we assume the 1.6% postcard recruitment rate from mailing postcards to the 61,101 adults who used mental health services in Victoria (in 2019-2020), resulting in an estimated 978 per in the first year (and assume this continues annually).  Thus, the annual cost of training per recruited client would be ($1,920 + $12)/978 = $1.98 per client recruited. | Australian Psychology Society: <https://psychology.org.au/>  Department of Health Victoria: <https://www.health.vic.gov.au/priorities-and-transformation/victorias-mental-health-services-annual-report-2019-20> |
| Quitline call-related costs | Quitline staff time was assumed to be AU$79.90 (AU$69.48 plus 15% on costs). The time of each call plus 40 minutes preparation and post call time per successful call and 5 minutes per unsuccessful attempt was included. An additional cost of 0.04c/mins plus AU$0.20 per call was added for telecommunications charges. | Quitline’s project records and standard Quitline pay scales for telephone counsellors including on-costs. |
| Postcard recruitment cost | We calculate the costs of printing and posting 8210 postcards (2 mail-outs approx. 6 months apart) that went to an estimated 4210 unique adult individuals (the currency of all addresses was not known) addresses of adults registered with two participating mental health service provider organisations, note their smoking status was unknown. This yielded 66 recruits. The cost per postcard printed was $0.10. Addressing and posting was performed by a mail out company at a cost of $0.62 per postcard. Total postcard costs were $0.72 x 8210=$5,911. The average cost per successful recruit was $5,911/66 = $89.56 | Study project records. |
| NRT costs | NRT costs were AU$23,380.21 in total for all 54 participants randomised to Quitlink and AU$17,457.85 for 42 who completed at least one of 5 months or 8 months follow-up (Table 3). Subsequent NRT costs were not included as there were no difference in NRT beyond 2-months (Table 5). | Study project records. |

**Table 3. Summary of NRT Costs for Quitlink intervention arm**

|  |  | **All (N=54)** | | **Complete 8m (N=42)** | |
| --- | --- | --- | --- | --- | --- |
| **NRT Type** | **Unit Cost** | **Total units** | **Total cost** | **Total units** | **Total cost** |
| Standard Patch (21mgx28) | $48.00 | 97 | $4,656.00 | 74 | $3,552.00 |
| Sensitive Patch (21mgx7) | $21.00 | 20 | $420.00 | 12 | $252.00 |
| Inhalators | $31.99 | 252 | $8,061.48 | 176 | $5,630.24 |
| Quickmist | $42.99 | 111 | $4,771.89 | 88.5 | $3,804.62 |
| Lozenge | $29.99 | 66 | $1,979.34 | 56 | $1,679.44 |
| Gum | $23.00 | 76 | $1,748.00 | 54 | $1,242.00 |
| Postage | $15.85 | 110 |  | 83 | $1,315.55 |
| TOTAL |  |  | $23,380.21 |  | $17,475.85 |

**Table 4. Self-reported hospital emergency department and inpatient service use**

|  | ***Control (N=47)*** | | ***Tx (N=37)*** | | ***Test for difference*** |
| --- | --- | --- | --- | --- | --- |
|  | Mean | SD | Mean | SD | p |
| Ambulance 0-8mths | .23 | .43 | .24 | .43 | 0.92 |
| ED - Mental 0-8mths | .21 | .41 | .03 | .16 | 0.01 |
| ED - Physical 0-8mths | .15 | .36 | .19 | .40 | 0.63 |
| Any Emergency 0-8mths | .30 | .46 | .35 | .48 | 0.61 |
| Hospital Mental 0-8mths | .21 | .41 | .11 | .31 | 0.19 |
| Hospital Physical 0-8mths | .19 | .40 | .22 | .42 | 0.78 |
| Community care unit 0-8mths | .00 | .00 | .03 | .16 | 0.32 |
| Preventative and recovery care centre 0-8mths | .11 | .31 | .11 | .31 | 0.98 |
| Other overnight admission 0-8mths | .04 | .20 | .05 | .23 | 0.81 |
| Any Admission 0-8mths | .38 | .49 | .30 | .46 | 0.41 |

**Table 5. Differences in subsidised Medicare Benefits Scheme (MBS) and Pharmaceutical Benefits Scheme (PBS) usage prior to randomisation & 8 months post.**

|  | **Control-pre** | | **Tx-pre** | | ***Delta*** | ***Test for difference*** | **Control-post** | | **Tx-post** | | ***Delta*** | ***Test for difference*** |  |
| --- | --- | --- | --- | --- | --- | --- | --- | --- | --- | --- | --- | --- | --- |
|  | Mean/Prop. | SD | Mean/Prop. | SD | *(Tx-control)* | *P>\|t\|* | Mean/Prop. | SD | Mean/Prop. | SD | *(Tx-control)* | *P>\|t\|* |  |
| Concessional card holder | 0.90 | 0.30 | 0.67 | 0.48 | *-0.24* | 0.05 | 0.90 | 0.30 | 0.67 | 0.48 | *-0.24* | 0.05 |  |
| ***Medicare Benefits Scheme (MBS) outcomes (Government subsidised health services)*** | | | | | | | | | | | | | |
| ***All MBS services*** | | | | | | | | | | | | | |
| Government benefit paid $AU | 251.90 | 282.94 | 178.86 | 166.33 | *-73.03* | 0.28 | 223.35 | 221.03 | 196.48 | 203.07 | *-26.86* | 0.66 |  |
| Out-of-pocket $AU | 2.26 | 7.62 | 19.82 | 46.54 | *17.56* | 0.08 | 1.47 | 5.99 | 18.29 | 42.89 | *16.81* | 0.07 |  |
| No. MBS services used | 4.58 | 4.49 | 3.24 | 2.73 | *-1.34* | 0.22 | 3.99 | 3.35 | 3.40 | 2.99 | *-0.59* | 0.52 |  |
| ***Mental health related services*** |  |  |  |  |  |  |  |  |  |  |  |  |  |
| Government benefit paid $AU | 43.88 | 78.69 | 48.49 | 72.51 | *4.60* | 0.83 | 43.80 | 92.21 | 61.67 | 139.38 | *17.87* | 0.61 |  |
| Out-of-pocket $AU | 0.00 | 0.00 | 6.27 | 21.41 | *6.27* | 0.17 | 0.00 | 0.00 | 6.71 | 32.20 | *6.71* | 0.32 |  |
| No. MBS services used | 0.39 | 0.62 | 0.47 | 0.63 | *0.08* | 0.65 | 0.33 | 0.78 | 0.46 | 0.79 | *0.14* | 0.55 |  |
|  |  |  |  |  |  |  |  |  |  |  |  |  |  |
| ***Pharmaceutical Benefits Scheme (PBS) outcomes (Government subsidised pharmaceuticals)*** | | | | | | | | | | | | | |
| ***All PBS use*** | | | | | | | | | | | | | |
| Government benefit paid $AU | 150.56 | 187.45 | 347.03 | 580.17 | *196.46* | 0.12 | 145.59 | 136.36 | 284.43 | 459.74 | *138.84* | 0.17 |  |
| Out-of-pocket $AU | 21.82 | 12.61 | 35.45 | 34.83 | *13.63* | 0.08 | 20.01 | 11.16 | 31.90 | 33.62 | *11.89* | 0.11 |  |
| No. scripts. | 4.13 | 3.64 | 3.98 | 4.11 | *-0.15* | 0.90 | 4.32 | 4.09 | 3.13 | 2.16 | *-1.19* | 0.24 |  |
| ***PBS use: nervous system related*** | | | | | | | | | | | | | |
| Government benefit paid $AU | 117.03 | 177.15 | 153.01 | 213.79 | *35.98* | 0.54 | 116.52 | 118.02 | 133.19 | 177.47 | *16.67* | 0.71 |  |
| Out-of-pocket $AU | 14.94 | 9.53 | 22.58 | 29.75 | *7.64* | 0.24 | 12.91 | 7.62 | 22.84 | 32.71 | *9.93* | 0.16 |  |
| No. scripts. | 2.65 | 2.07 | 2.09 | 1.92 | *-0.56* | 0.35 | 2.73 | 2.61 | 1.78 | 1.25 | *-0.96* | 0.13 |  |
| ***PBS use: smoking cessation related*** | | | | | | | | | | | | | |
| Government benefit paid $AU | 6.22 | 13.80 | 0.85 | 3.62 | *-5.37* | 0.09 | 6.47 | 15.26 | 2.81 | 7.93 | *-3.66* | 0.33 |  |
| Out-of-pocket $AU | 0.78 | 1.63 | 0.52 | 2.07 | *-0.26* | 0.64 | 0.62 | 1.62 | 0.21 | 0.61 | *-0.42* | 0.27 |  |
| No. scripts. | 0.14 | 0.30 | 0.03 | 0.09 | *-0.11* | 0.11 | 0.14 | 0.33 | 0.04 | 0.10 | *-0.10* | 0.19 |  |
| **Notes:** All outcomes presented are averaged at the monthly level. Individuals were observed on average 21.4 months, before randomisation. Post-randomisation period is 8 months. MBS outcomes exclude inpatient services. 25 and 21 people in the CONTROL group consented to MBS and PBS respectively. 23 and 24 people consented to the MBS and PBS respectively. Tests for difference calculated using t-test and clustered at the individual level. | | | | | | | | | | | | |  |

**References**

1. Sweeney R, Moodie M, Baker AL, Borland R, Castle D, Segan C, Turner A, Attia J, Kelly PJ, Brophy L, Bonevski B, Williams JM, Baird D, White SL and McCarter K. Protocol for an economic evaluation of the quitlink randomized controlled trial for accessible smoking cessation support for people with severe mental illness. Clinical Study Protocol. Frontiers in Psychiatry. 2019-September-03 2019;10doi:10.3389/fpsyt.2019.00618

2. Baker AL, McCarter K, Brophy L, Castle D, Kelly PJ, Cocks N, McKinlay ML, Brasier C, Borland R, Bonevski B, Segan C, Baird DE, Turner A, Williams JM, Forbes E, Hayes L, Attia J, Lambkin D, Barker D and Sweeney R. Adapting peer researcher facilitated strategies to recruit people receiving mental health services to a tobacco treatment trial. Front Psychiatry. 2022;13:869169. doi:10.3389/fpsyt.2022.869169

3. Richardson J, Iezzi A, Khan MA and Maxwell A. Validity and reliability of the assessment of quality of life (aqol)-8d multi-attribute utility instrument. The Patient - Patient-Centered Outcomes Research. 2014/03/01 2014;7(1):85-96. doi:10.1007/s40271-013-0036-x

4. Chen G and Olsen JA. Filling the psycho-social gap in the eq-5d: The empirical support for four bolt-on dimensions. Quality of life research : an international journal of quality of life aspects of treatment, care and rehabilitation. 2020;29(11):3119-3129. doi:10.1007/s11136-020-02576-5

5. Bradley Efron RJT. An introduction to the bootstrap. Chapman & Hall; 1993.

6. Royal Commission into Victoria’s Mental Health System. Final report: Summary and recommendations [available from: <Https://finalreport.Rcvmhs.Vic.Gov.Au/download-report/>]. 2021.

7. Barnett PG, Wong W and Hall S. The cost-effectiveness of a smoking cessation program for out-patients in treatment for depression. <https://doi.org/10.1111/j.1360-0443.2008.02167.x>. Addiction. 2008/05/01 2008;103(5):834-840. doi:<https://doi.org/10.1111/j.1360-0443.2008.02167.x>

8. Wang S, Gum D and Merlin T. Comparing the icers in medicine reimbursement submissions to nice and pbac-does the presence of an explicit threshold affect the icer proposed? Value Health. Aug 2018;21(8):938-943. doi:10.1016/j.jval.2018.01.017

9. Saxby K, Ireland A, Ghijben P, Sweeney R, Sia K-L, Chen E, Farrell M, McRobbie H, Courtney R and Petrie D. Household composition, smoking cessation and relapse: Results from a prospective longitudinal australian cohort. medRxiv. 2022:2022.01.03.22268695. doi:10.1101/2022.01.03.22268695

10. Courtney RJ, McRobbie H, Tutka P, Weaver NA, Petrie D, Mendelsohn CP, Shakeshaft A, Talukder S, Macdonald C, Thomas D, Kwan BCH, Walker N, Gartner C, Mattick RP, Paul C, Ferguson SG, Zwar NA, Richmond RL, Doran CM, Boland VC, Hall W, West R and Farrell M. Effect of cytisine vs varenicline on smoking cessation: A randomized clinical trial. Jama. Jul 6 2021;326(1):56-64. doi:10.1001/jama.2021.7621

11. Thun MJ, Hannan LM, Adams-Campbell LL, Boffetta P, Buring JE, Feskanich D, Flanders WD, Jee SH, Katanoda K, Kolonel LN, Lee IM, Marugame T, Palmer JR, Riboli E, Sobue T, Avila-Tang E, Wilkens LR and Samet JM. Lung cancer occurrence in never-smokers: An analysis of 13 cohorts and 22 cancer registry studies. PLoS Med. Sep 30 2008;5(9):e185. doi:10.1371/journal.pmed.0050185

12. Pesch B, Kendzia B, Gustavsson P, Jöckel KH, Johnen G, Pohlabeln H, Olsson A, Ahrens W, Gross IM, Brüske I, Wichmann HE, Merletti F, Richiardi L, Simonato L, Fortes C, Siemiatycki J, Parent ME, Consonni D, Landi MT, Caporaso N, Zaridze D, Cassidy A, Szeszenia-Dabrowska N, Rudnai P, Lissowska J, Stücker I, Fabianova E, Dumitru RS, Bencko V, Foretova L, Janout V, Rudin CM, Brennan P, Boffetta P, Straif K and Brüning T. Cigarette smoking and lung cancer--relative risk estimates for the major histological types from a pooled analysis of case-control studies. Int J Cancer. Sep 1 2012;131(5):1210-9. doi:10.1002/ijc.27339

13. Afonso AS, Verhamme KM, Sturkenboom MC and Brusselle GG. Copd in the general population: Prevalence, incidence and survival. Respir Med. Dec 2011;105(12):1872-84. doi:10.1016/j.rmed.2011.06.012

14. Bhatt SP, Kim YI, Harrington KF, Hokanson JE, Lutz SM, Cho MH, DeMeo DL, Wells JM, Make BJ, Rennard SI, Washko GR, Foreman MG, Tashkin DP, Wise RA, Dransfield MT and Bailey WC. Smoking duration alone provides stronger risk estimates of chronic obstructive pulmonary disease than pack-years. Thorax. May 2018;73(5):414-421. doi:10.1136/thoraxjnl-2017-210722

15. van Durme Y, Verhamme KMC, Stijnen T, van Rooij FJA, Van Pottelberge GR, Hofman A, Joos GF, Stricker BHC and Brusselle GG. Prevalence, incidence, and lifetime risk for the development of copd in the elderly: The rotterdam study. Chest. Feb 2009;135(2):368-377. doi:10.1378/chest.08-0684

16. Chang JT, Meza R, Levy DT, Arenberg D and Jeon J. Prediction of copd risk accounting for time-varying smoking exposures. PLoS One. 2021;16(3):e0248535. doi:10.1371/journal.pone.0248535

17. Spencer M, Briggs AH, Grossman RF and Rance L. Development of an economic model to assess the cost effectiveness of treatment interventions for chronic obstructive pulmonary disease. Pharmacoeconomics. 2005;23(6):619-37. doi:10.2165/00019053-200523060-00008

18. Quanjer PH, Tammeling GJ, Cotes JE, Pedersen OF, Peslin R and Yernault J-C. Lung volumes and forced ventilatory flows. European Respiratory Journal. 1993;6(Suppl 16):5-40. doi:10.1183/09041950.005s1693

19. Banks E, Joshy G, Korda RJ, Stavreski B, Soga K, Egger S, Day C, Clarke NE, Lewington S and Lopez AD. Tobacco smoking and risk of 36 cardiovascular disease subtypes: Fatal and non-fatal outcomes in a large prospective australian study. BMC Medicine. 2019/07/03 2019;17(1):128. doi:10.1186/s12916-019-1351-4

20. Lubin JH, Couper D, Lutsey PL, Woodward M, Yatsuya H and Huxley RR. Risk of cardiovascular disease from cumulative cigarette use and the impact of smoking intensity. Epidemiology. May 2016;27(3):395-404. doi:10.1097/ede.0000000000000437

21. Rea TD, Heckbert SR, Kaplan RC, Smith NL, Lemaitre RN and Psaty BM. Smoking status and risk for recurrent coronary events after myocardial infarction. Ann Intern Med. Sep 17 2002;137(6):494-500. doi:10.7326/0003-4819-137-6-200209170-00009

22. Islam MS, Anderson CS, Hankey GJ, Hardie K, Carter K, Broadhurst R and Jamrozik K. Trends in incidence and outcome of stroke in perth, western australia during 1989 to 2001: The perth community stroke study. Stroke. Mar 2008;39(3):776-82. doi:10.1161/strokeaha.107.493643

23. Chen J, Li S, Zheng K, Wang H, Xie Y, Xu P, Dai Z, Gu M, Xia Y, Zhao M, Liu X and Xu G. Impact of smoking status on stroke recurrence. J Am Heart Assoc. Apr 16 2019;8(8):e011696. doi:10.1161/jaha.118.011696

24. Flach C, Muruet W, Wolfe CDA, Bhalla A and Douiri A. Risk and secondary prevention of stroke recurrence: A population-base cohort study. Stroke. Aug 2020;51(8):2435-2444. doi:10.1161/strokeaha.120.028992

25. Australian Institute of Health and Welfare. General record of incidence of mortality 2019. 2019. Available from: <Https://www.Aihw.Gov.Au/reports/life-expectancy-deaths/grim-books/contents/grim-excel-workbooks> (accessed december 2020).

26. Cadilhac DA, Kilkenny MF, Levi CR, Lannin NA, Thrift AG, Kim J, Grabsch B, Churilov L, Dewey HM, Hill K, Faux SG, Grimley R, Castley H, Hand PJ, Wong A, Herkes GK, Gill M, Crompton D, Middleton S, Donnan GA and Anderson CS. Risk-adjusted hospital mortality rates for stroke: Evidence from the australian stroke clinical registry (auscr). Medical Journal of Australia. 2017;206(8):345-350. doi:<https://doi.org/10.5694/mja16.00525>

27. Pharmaceutical Benefits Scheme. Post-market review of medicines for smoking cessation: Report to the pharmaceutical benefits advisory committee. Tor 4: Cost-effectiveness review of specified combinations of smoking cessation medicines and estimates for the pharmaceutical benefits scheme. (forthcoming). <Https://www.Pbs.Gov.Au/info/reviews/post-market-review-of-medicines-for-smoking-cessation>
