# Supplementary material for: ‘Quitlink’: Outcomes of a randomised controlled trial of peer researcher facilitated referral to a tailored quitline tobacco treatment for people receiving mental health services
Source: Aust N Z J Psychiatry. 2023 Jun 23;58(3):260–76. doi: 10.1177/00048674231181039 (PMC10903138; doi:10.1177/00048674231181039)
Supplement: sj-docx-2-anp-10.1177_00048674231181039 – Supplemental material for ‘Quitlink’: Outcomes of a randomised controlled trial of peer researcher facilitated referral to a tailored quitline tobacco treatment for people receiving mental health services [file sj-docx-2-anp-10.1177_00048674231181039.docx]

Supplementary Table 1 Selected key participant characteristics according to 8-month follow-up completion status

| Variable | Did not complete 8-month follow-up  (n=25) (n, %) | Completed 8-month follow-up  (n=84) (n,%) | Total  (N=109) (n, %) |
| --- | --- | --- | --- |
| Gender (female) | 13 (52.0) | 43 (51.2) | 56 (51.4) |
| Age (Mean years, SD) | 40.13 (14.26) | 46.51 (12.30) | 44.89 (13.05) |
| Married / defacto | 4 (16.0) | 12 (14.3) | 16 (14.7) |
| Unemployed | 17 (68.0) | 47 (56.0) | 64 (58.7) |
| Left school at or before 16 years | 9 (36.0) | 24 (28.6) | 35 (32.1) |
| Recruitment Clinic Type  Residential  Community based | 10 (40.0)  15 (60.0) | 15 (17.9)  69 (82.0) | 25 (22.9)  84 (77.0) |
| 7-day point prevalence abstinence (yes)  2-months  5-months | 2 (11.0) (n=18)  1 (14.3) (n=7) | 11 (14.0) (n=81)  15 (19.0) (n=79.5) | 13 (13.1) (n=99)  16 (18.4) (n=87) |
| Cigarettes per day (daily smokers) (Mean, SD)  Baseline  2-months  5-months | 20.48 (10.39)  12.71 (11.22) (n=17)  13.86 (12.65) (n=7) | 20.89 (9.61)  14.25 (12.73) (n=75)  13.55 (13.71) (n=76) | 20.80 (9.75)  13.97 (12.43) (n=92)  13.58 (13.55) (n=83) |
| HSI Addiction Category  Baseline  Low  Moderate  High  2-months  Quit Smoking  Low  Moderate  High  5-months  Quit Smoking  Low  Moderate  High | 5 (24.0)  14 (56.0)  6 (24.0)  (n=17)  2 (11.8)  7 (41.2)  6 (35.3)  2 (11.8)  (n=7)  1 (14.3)  3 (42.9)  1 (14.3)  2 (28.6) | 8 (9.5)  58 (69.0)  18 (21.4)  (n=80)  12 (15.0)  30 (37.5)  28 (35.0)  10 (12.5)  (n=80)  17 (21.3)  26 (32.5)  30 (37.5)  7 (8.8) | 13 (11.9%)  72 (66.1%)  24 (22.0%)  (n= 97)  14 (14.4)  37 (38.1)  34 (35.1)  12 (12.4)  (n=87)  18 (20.7)  29 (33.3)  31 (35.6)  9 (10.3) |
| NRT Use (% in previous month)  Baseline  2-months  5-months | 17 (68.0)  11 (64.7) (n=17)  3 (42.9) (n=7) | 66 (78.6)  57 (70.4) (n=81)  46 (57.5) (n=80) | 83 (76.1)  68 (69.4) (n=98)  49 (56.3) (n=87) |
| MINI diagnosis Psychotic disorder  Other diagnosis  Missing diagnosis | 8 (80.0)  2 (20.0)  15 | 51 (66.2)  26 (33.8)  7 | 59 (67.8)  28 (32.2)  22 |

HSI: Heaviness of Smoking Index^1, 2^; MINI: Mini International Neuropsychiatric Interview^3^; NRT: nicotine replacement therapy

1. Heatherton TF, Kozlowski LT, Frecker RC, Rickert W and Robinson J. Measuring the heaviness of smoking: Using self-reported time to the first cigarette of the day and number of cigarettes smoked per day. British Journal of Addiction. 1989;84(7):791-800. doi:<https://doi.org/10.1111/j.1360-0443.1989.tb03059.x>

2. Kozlowski LT, Porter CQ, Orleans CT, Pope MA and Heatherton T. Predicting smoking cessation with self-reported measures of nicotine dependence: Ftq, ftnd, and hsi. Drug and Alcohol Dependence. 1994/02/01/ 1994;34(3):211-216. doi:<https://doi.org/10.1016/0376-8716(94)90158-9>

3. Sheehan DV, Lecrubier Y, Sheehan KH, Amorim P, Janavs J, Weiller E, Hergueta T, Baker R and Dunbar GC. The mini-international neuropsychiatric interview (mini): The development and validation of a structured diagnostic psychiatric interview for dsm-iv and icd-10. Journal of clinical psychiatry. 1998;59(20):22-33.
