# Supplementary material for: ‘Quitlink’: Outcomes of a randomised controlled trial of peer researcher facilitated referral to a tailored quitline tobacco treatment for people receiving mental health services
Source: Aust N Z J Psychiatry. 2023 Jun 23;58(3):260–76. doi: 10.1177/00048674231181039 (PMC10903138; doi:10.1177/00048674231181039)
Supplement: sj-docx-3-anp-10.1177_00048674231181039 – Supplemental material for ‘Quitlink’: Outcomes of a randomised controlled trial of peer researcher facilitated referral to a tailored quitline tobacco treatment for people receiving mental health services [file sj-docx-3-anp-10.1177_00048674231181039.docx]

Supplementary Table 2 Additional secondary outcomes

| Variable | Assessment Occasion | Control  Mean (SD) (N=55) | Quitlink  Mean (SD) (N=54) | LS Mean Difference (N=109)  (intention to treat analysis) | p-value |
| --- | --- | --- | --- | --- | --- |
| AQOL Utility Score | Baseline | 0.496 (0.196) | 0.549 (0.196) |  |  |
|  | 2-months | 0.577 (0.219) | 0.570 (0.211) | 0.05 (-0.04, 0.14) | 0.251 |
|  | 5-months | 0.561 (0.195) | 0.610 (0.213) | 0.05 (-0.03, 0.14) | 0.229 |
|  | 8-months | 0.590 (0.195) | 0.640 (0.215) | 0.05 (-0.03, 0.13) | 0.178 |
| K10 Category | Baseline  Well  Mild  Moderate  Severe | (n=54)  10 (18.5)  7 (13.0)  10 (18.5)  27 (50.0) | (n=49)  10 (20.4)  14 (28.6)  10 (20.4)  15 (30.6) | OR (95% CI) between conditions (N=106)  Available case |  |
|  | 2-months  Well  Mild  Moderate  Severe | n=46  8 (17.4)  6 (13.0)  12 (26.1)  20 (43.0) | n=43  8 (18.6)  10 (23.3)  10 (23.3)  15 (34.9) | 0.60 (0.19, 1.85) | 0.37 |
|  | 5-months  Well  Mild  Moderate  Severe | n=45  13 (29.0)  8 (17.8)  5 (11.1)  19 (42.2) | n=32  8 (25.0)  9 (28.1)  8 (25.0)  7 (22.0) | 0.79 (0.24, 2.60) | 0.699 |
|  | 8-months  Well  Mild  Moderate  Severe | n=45  13 (28.9)  8 (18.0)  11 (24.4)  13 (28.9) | n=30  10 (33.0)  8 (26.7)  7 (23.3)  5 (16.7) | 0.51 (0.15, 1.72) | 0.277 |
| AUDIT-C risky drinking | Baseline | n=54  22 (40.1) | n=53  28 (52.8) | OR (95% CI) between conditions (N=109)  Available case |  |
|  | 2-months | n=49  17 (34.7) | n=46  20 (37.7)) | 1.72 (0.34, 8.81) | 0.511 |
|  | 5-months | n=46  18 (39.1) | n=40  13 (32.5) | 0.85 (0.16, 4.59) | 0.847 |
|  | 8-months | n=46  20 (43.5) | n=36  14 (38.9) | 0.70 (0.12, 3.89) | 0.678 |

ITT: Intention to treat; AQOL: Australian Quality of Life Index; AUDIT Alcohol Use Disorder Identification Test ^1^

1. Bush K, Kivlahan DR, McDonell MB, Fihn SD, Bradley KA and for the Ambulatory Care Quality Improvement Project. The audit alcohol consumption questions (audit-c): An effective brief screening test for problem drinking. Archives of Internal Medicine. 1998;158(16):1789-1795. doi:10.1001/archinte.158.16.1789
