# Supplementary material for: ‘Quitlink’: Outcomes of a randomised controlled trial of peer researcher facilitated referral to a tailored quitline tobacco treatment for people receiving mental health services
Source: Aust N Z J Psychiatry. 2023 Jun 23;58(3):260–76. doi: 10.1177/00048674231181039 (PMC10903138; doi:10.1177/00048674231181039)
Supplement: sj-docx-4-anp-10.1177_00048674231181039 – Supplemental material for ‘Quitlink’: Outcomes of a randomised controlled trial of peer researcher facilitated referral to a tailored quitline tobacco treatment for people receiving mental health services [file sj-docx-4-anp-10.1177_00048674231181039.docx]

Supplementary Table 3 Psychiatric adverse events

|  | **Control** | | **Intervention** | |
| --- | --- | --- | --- | --- |
| **Adverse Event Description** | **Yes AE^*^** | **No AE^**^** | **Yes AE^*^** | **No AE^**^** |
| *Depression and/or anxiety^a^* | *12* | *43* | *18* | *36* |
| Depression | 10 | 45 | 8 | 46 |
| Anxiety | 3 | 52 | 6 | 48 |
| Psychiatric decompensation | 5 | 50 | 3 | 51 |
| Anxiety/depression | 1 | 54 | 5 | 49 |
| Stress | 3 | 52 | 2 | 52 |
| Suicidal ideation | 1 | 54 | 3 | 51 |
| Suicide attempt | 3 | 52 | 1 | 53 |
| Mania | 2 | 53 | 1 | 53 |
| Nightmares | 3 | 52 | 0 | 54 |
| Intentional self injury | 2 | 53 | 1 | 53 |
| Sleep disorder | 1 | 54 | 2 | 52 |
| Panic attacks | 1 | 54 | 1 | 53 |
| Post-traumatic stress disorder | 0 | 55 | 2 | 52 |
| Substance use | 2 | 53 | 0 | 54 |
| Psychosis | 2 | 53 | 0 | 54 |
| Auditory hallucinations | 0 | 55 | 1 | 53 |
| Cognitive disorder | 0 | 55 | 1 | 53 |
| Drug-induced psychosis | 0 | 55 | 1 | 53 |
| Irritability | 1 | 54 | 0 | 54 |
| Restlessness | 1 | 54 | 0 | 54 |
| Schizoaffective disorder | 0 | 55 | 1 | 53 |
| Schizophrenia | 0 | 55 | 1 | 53 |
| Teeth grinding | 0 | 55 | 1 | 53 |
| Vivid dreams | 0 | 55 | 1 | 53 |

Notes: ^*^ Number of individual participants who reported the adverse event during the trial. ^**^ Number of individual participants who did not report the adverse event during the trial. ^a^ Number of individual participants who reported depression, anxiety and/or depression/anxiety during the trial.
